# Induction of Enhanced Acoustic Startle Response by Noise Exposure: Dependence on Exposure Conditions and Testing Parameters and Possible Relevance to Hyperacusis

**DOI:** 10.1371/journal.pone.0111747

**Published:** 2014-10-31

**Authors:** Rony H. Salloum, Christopher Yurosko, Lia Santiago, Sharon A. Sandridge, James A. Kaltenbach

**Affiliations:** 1 Department of Neurosciences, The Cleveland Clinic, Cleveland, Ohio, United States of America; 2 Head and Neck Institute, The Cleveland Clinic, Cleveland, Ohio, United States of America; University of California, Irvine, United States of America

## Abstract

There has been a recent surge of interest in the development of animal models of hyperacusis, a condition in which tolerance to sounds of moderate and high intensities is diminished. The reasons for this decreased tolerance are likely multifactorial, but some major factors that contribute to hyperacusis are increased loudness perception and heightened sensitivity and/or responsiveness to sound. Increased sound sensitivity is a symptom that sometimes develops in human subjects after acoustic insult and has recently been demonstrated in animals as evidenced by enhancement of the acoustic startle reflex following acoustic over-exposure. However, different laboratories have obtained conflicting results in this regard, with some studies reporting enhanced startle, others reporting weakened startle, and still others reporting little, if any, change in the amplitude of the acoustic startle reflex following noise exposure. In an effort to gain insight into these discrepancies, we conducted measures of acoustic startle responses (ASR) in animals exposed to different levels of sound, and repeated such measures on consecutive days using a range of different startle stimuli. Since many studies combine measures of acoustic startle with measures of gap detection, we also tested ASR in two different acoustic contexts, one in which the startle amplitudes were tested in isolation, the other in which startle amplitudes were measured in the context of the gap detection test. The results reveal that the emergence of chronic hyperacusis-like enhancements of startle following noise exposure is highly reproducible but is dependent on the post-exposure thresholds, the time when the measures are performed and the context in which the ASR measures are obtained. These findings could explain many of the discrepancies that exist across studies and suggest guidelines for inducing in animals enhancements of the startle reflex that may be related to hyperacusis.

## Introduction

Hyperacusis is a condition characterized by a heightened sensitivity to sounds and manifesting as diminished sound tolerance [1]–[4], but can include increased loudness perception or increased responsiveness to sound [5]. Hyperacusis is a common result of acoustic trauma and is frequently seen in association with tinnitus. The incidence of hyperacusis in the general population is unknown, but estimates range from 1 to 22% [6]–[8]. A large percentage (40–80%) of subjects with tinnitus also suffer from hyperacusis [1], [3], [9], [10]. Like tinnitus, hyperacusis occurs in acute and chronic forms. Acute hyperacusis is experienced for short periods ranging from minutes to weeks, while chronic hyperacusis lasts many weeks, months or years. These different clinical features suggest that the mechanisms underlying hyperacusis may be complex and may share some characteristics with those underlying tinnitus. However, the ability to test this hypothesis and separate those that are linked to tinnitus and those linked to hyperacusis requires the development of well-defined animal models of hyperacusis in its acute and chronic forms.

A growing number of studies have demonstrated induction of acute or chronic increases in responsiveness to sound that could be related to hyperacusis. Ison and colleagues found that mice show an augmentation of the acoustic startle reflex (ASR) with age [11]. Turner and Parrish [12] reported that the suppression of the ASR by a preceding pulse of sound (pre-pulse inhibition) was enhanced in rats treated with sodium salicylate, a finding which they interpreted as suggestive of hyperacusis. Two studies from the laboratory of Sun [13], [14] found evidence for acute enhancements of the acoustic startle response (ASR) in rats treated with sodium salicylate. Subsequently, a transient strengthening of pre-pulse inhibition was also found in mice that had been exposed to intense noise [15]. Sun and colleagues [16] observed transient enhancements of the ASR in rats previously exposed to noise, while Dehmel et al. [17] found chronic enhancements of absolute startle amplitude and enhanced pre-pulse inhibition in noise-exposed guinea pigs. Thus, aging, salicylate treatment and noise exposure all appear to be factors that can trigger induction of heightened responsiveness to sound, although in most cases, the changes were found to be weak and very transient.

Recently, our laboratory showed that exposure to intense sound can lead to the induction of robust and long lasting enhancements of the ASR [18]. The enhancements were observed in the range of weeks to months following sound exposure and were associated with enhancements of noise-induced suppression of startle. Qualitatively similar chronic enhancements of the ASR and prepulse inhibition were observed in mice following moderate sound exposure [19], and such changes were found to be associated with cochlear neuropathy [20]. However, the results across studies have not always been consistent. While the study by Hickox and Liberman [19] showed enhancements of startle when the startle stimulus was immediately preceded by continuous background noise, only a slight suggestion of enhanced startle was observed when the startle stimulus was presented without background noise. Moreover, some studies have reported noise-induced changes in auditory responses that are not consistent with the above described enhancements of startle. Longenecker and Galazyuk [21] presented data showing little if any change in the ASR in noise exposed mice at or slightly above startle threshold, and startle amplitudes at moderate to high levels of startle stimulation were lower than control levels. Similarly, the data of Lobarinas et al. [22] showed a consistent weakening of startle amplitude at almost all startle stimulus levels above startle thresholds.

The different effects of noise exposure on the acoustic startle reflex across studies raise the question of what factors might be critical in determining whether a noise exposure condition or a condition of testing leads to enhancement of startle responses. In an effort to gain insight into these issues, we explored in depth how the acoustic startle response changes with variations in a number of different parameters. In particular, we sought to determine whether the induction of such enhancements is dependent on the intensity of exposure, and if so, what the optimal exposure condition might be to maximally induce enhanced startle responses. In order to understand the temporal nature of enhanced startle, we measured ASR at different post-exposure recovery times. Finally, we tested whether the changes in ASR might depend on the context of the measures. In particular, we examined the effect of performing ASR measures alone or in the context of the gap detection test. Our results suggest that all of these parameters are critical in determining whether enhancements of the ASR are induced and readily observable following sound exposure.

## Methods

### Ethical considerations

Animal work was performed using practices fully compliant with the NIH guidelines for the care and use of animals in research. Animals were lightly anesthetized by intramuscular injection of ketamine/xylazine (58 mg/kg- 9 mg/kg) for the measurements of Auditory Brainstem Responses. The animal handling protocol (#2013 1151) was approved by the Institutional Animal Care and Use Committee (IACUC) of the Cleveland Clinic.

### Animal subjects

Adult hamsters (LVG strain), 60–70 days of age at the time of arrival were maintained on a 12∶12-h day/night cycle by the animal housing facility of the Cleveland Clinic. After a 4-day quarantine period, they were divided into two groups, including an experimental group to be sound exposed, and a control group of unexposed animals. For each experiment, the exposed group was subdivided to allow testing of different variables. Exposures generally were conducted when the animals were between 2 and 3 months of age, except when noted otherwise. ASR tests were conducted beginning on each of the first three days after exposure, then every 2–3 days thereafter over an approximately 2 week period. ABRs were conducted upon completion of ASR tests.

### Sound exposure

The animals were exposed to sounds inside a cylindrical chamber placed inside an Acoustic Systems sound attenuation booth. The chamber contained 4 compartments, allowing exposure of up to 4 animals simultaneously. Sound was introduced into the chamber through a Beyma CP-25 speaker mounted in the lid of the cylinder. The exposure sound was a 10 kHz continuous tone, calibrated using an Etymotic ER7C probe tube microphone whose tip was placed 2 cm above the chamber’s floor to approximate the position where the animals ears would be located during the exposure. Since sound level varied when measurements were taken at different locations on the floor of the chamber, we chose a voltage input to the speaker that produced the desired sound level when averaged across those locations. Animals were allowed a few minutes of silence once placed inside the chamber to permit acclimatization to the exposure environment. The sound was then turned on and gradually increased in level over a 10 minute period before reaching the final exposure intensity. This approach was found to protect the animals from stress caused by sudden onset of intense sound. The exposure sound was delivered continuously for 4 hours. Behavior was monitored throughout the exposure period to ensure that the animals did not later develop behaviors indicative of stress. Exposures were performed in three different rounds, each differing in intensity level. Animals were exposed at 110 dB SPL in the first round, 115 dB SPL in the second, and at 120 dB SPL in the third. In each round, 5–8 animals were exposed to the tone, while 5–7 others were placed in the exposure chamber for a 4 hour period of silence and served as controls. Following the exposures the animals were returned to the animal facility. The acoustic startle reflex measurements began the following day in each trial.

### Acoustic startle apparatus

Measures of acoustic startle were recorded as in our previous publication [18]. The testing apparatus consisted of a Kinder Scientific startle system (Model SM100) consisting of a small chamber measuring 28×36×50 cm and containing a small animal housing designed for use with rats. The chamber was insulated with a 1 inch layer of dense foam material to reduce sound reflections [21]. Each animal was placed inside the housing on top of a plate that contained a pressure-sensitive piezoelectric transducer that converted sudden pressure changes caused by animal movement into voltages that were scaled to pressure units (Newtons, N). The plate sensitivity was adjusted to generate a 1±0.05 N on the first positive peak of the pressure pulse waveform in response to a 1.0 N calibration wave. Calibration of the sound level at the level of the animals’ ears was performed using a 1/4 in microphone and sound measurement system (B&K). Startle stimuli (and where applicable, stimuli used for the gap detection test) were introduced from speakers located in the ceiling of the test chamber. Inputs and measurements were controlled using Kinder software, with startle response collection windows spanning 100 ms from startle stimulus onset. Animals were weighed before ASR measurements to account for the potential contribution of weight differences to the startle amplitudes.

### Acoustic stimuli

Testing was performed by quantifying the startle amplitude as a function of the startle stimulus level. For that purpose, each startle measuring session consisted of five sets of 15 trials, each having a battery of twelve 20-ms bursts of broad band noise varied in level from 57–120 dB SPL in steps of 3–6 dB, plus three no-stimulus trials. The order of these stimuli and the duration of inter-trial intervals were randomized. The same session was repeated daily for each of the animal subgroups, so that all exposed and control animals were alternately tested on the same day.

**Figure 1:**
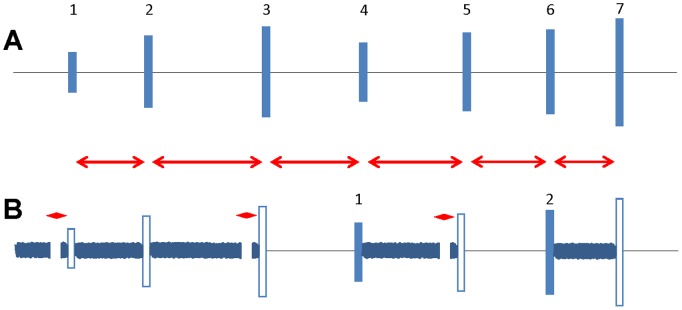
Stimulus sequences used to test the effect of acoustic context on ASR. Startle stimuli of different intensities (blue blocks) are presented as bursts separated by different time intervals (red double-headed arrows). In the first context (A), silence is maintained during the inter-stimulus interval (black lines) throughout the test battery. In the second context (B), a background noise, with or without an intervening gap of silence, fills some of the inter-stimulus intervals, but the startle stimulus only condition is identical to that in context 1. In either context, numbered solid blue block are those used to determine the ASR. Red diamond: time interval during which the background noise is interrupted by a gap.

Because the ASR is known to be sensitive to the presence of background noise [23]–[26], we tested whether ASR amplitudes might differ significantly depending on the context in which the startle stimuli were presented. In this experiment, startle eliciting stimuli were presented in two different ways. In the first method, ASRs were tested using a stimulus battery that included only startle-eliciting noise bursts presented randomly at different levels (Fig. 1A). In the second, ASRs were measured using the same startle-eliciting noise bursts that were used in the first method, except that these stimuli were inserted randomly in a battery of stimuli used to measure gap detection ability; this stimulus battery thus included startle eliciting stimuli preceded by background noise (with or without gaps of silence) as well as startle eliciting noise bursts not preceded by background noise (Fig. 1B). This experiment design allowed us to address the question of whether the startle amplitude evoked by identical stimuli (noise bursts) differs depending on the acoustic context (i.e., the presence or absence of a recent history of background noise in the test battery).

### Auditory brainstem responses (ABRs)

ABRs were recorded once behavioral testing was completed to assess the extent of hearing loss caused by sound exposure. This was generally a period of 3 to 5 weeks postexposure, but always just after the last ASR measurement. Each animal was lightly anesthetized by intramuscular injection of ketamine/xylazine (58 mg/kg- 9 mg/kg). Temperature was maintained at 37°C throughout the period of ABR testing. Needle electrodes were placed subcutaneously, one in in the vertex (non-inverting), one each behind the ears (inverting), and one in the right hind limb (ground). Electrode signals were amplified 100,000X and bandpass filtered (30–3000 Hz). Stimuli were tone pips varied in frequency from 4 to 16 kHz and presented at a rate of 17.7/s. The pips were presented at the highest levels (80–100 dB SPL) first then lowered in 20 dB steps until no responses were visible. At each level, responses were averaged over 250 stimulus repetitions. Two responses were obtained at each stimulus level to confirm presence or absence of a response. Intensity was bracketed in 5 dB steps in the range of the lowest levels of stimulation to determine threshold. ABR waveforms displayed 5–6 biphasic responses, and thresholds were based on measures of P4 or P5, whichever gave the lower threshold.

### Data analysis

Measures of startle amplitude were averaged for each animal, first across trials for each session then across sessions conducted on successive days. These were then averaged across animals for each of the stimulus levels tested. The result was a plot of startle amplitude vs. stimulus level for each animal. The mean startle amplitude vs. stimulus level (startle growth curve) was then obtained for each group by averaging across animals for each exposure or control group. Although control and exposed animals were age-matched, differences between growth curves in exposed and control animals could be due to group differences in mean weight. To control for this possibility, we averaged the weights across sessions to obtain a mean weight for each animal. Group weights were obtained by averaging the mean weights of all animals within each group. Group differences for all parameters of stimulation tested and for weights were tested using two-way ANOVAs or paired t-tests (one- or two-tailed), performed using Prism Graphpad. Differences between groups for each pairwise comparison were considered significant if P≤0.05.

## Results

### Effect of exposure intensity on response thresholds

**Figure 2:**
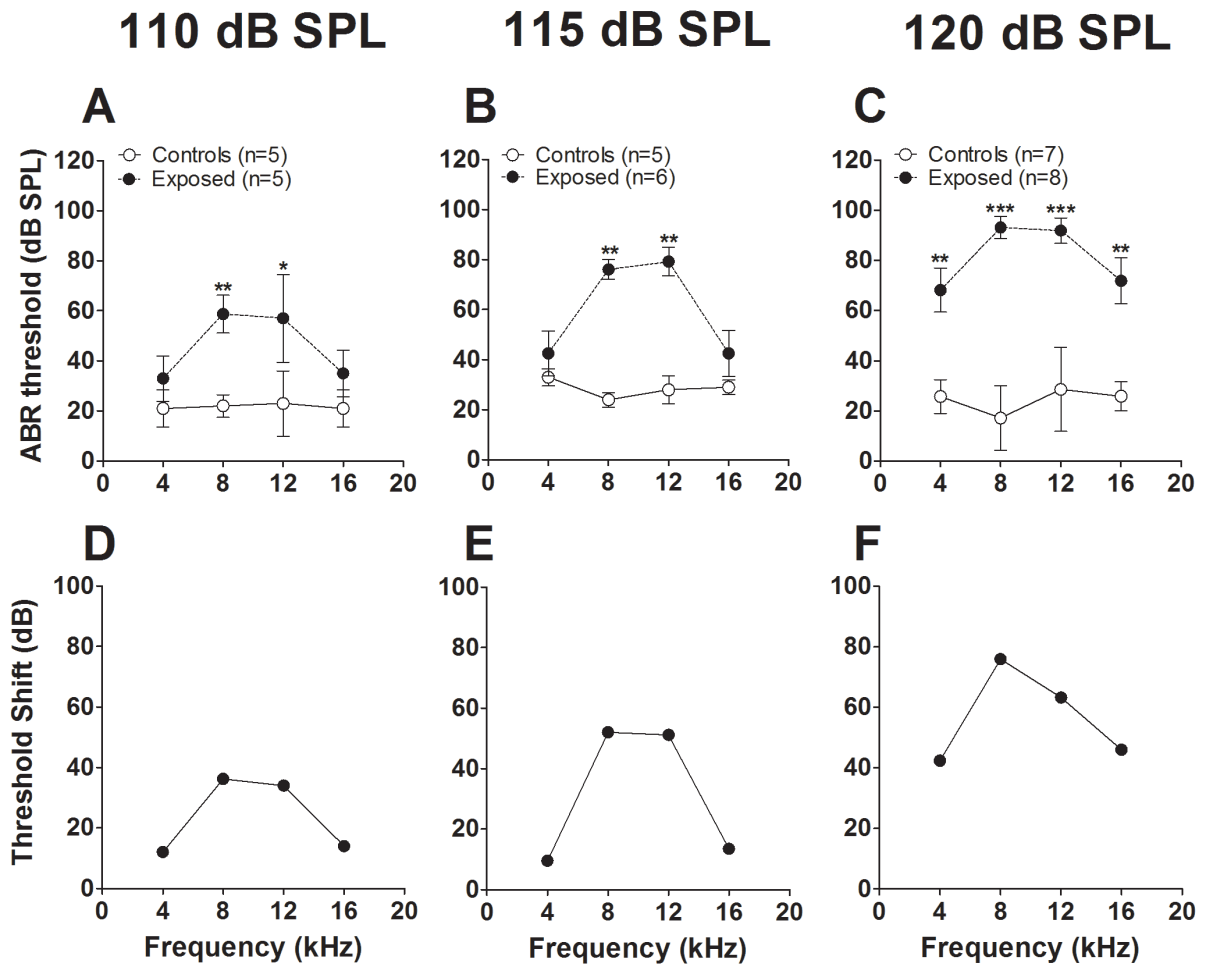
Effect of increasing the level of exposure on ABR thresholds. The exposure sound was a continuous 10 kHz tone presented for 4 hours at a level of 110 dB SPL (A), 115 dB SPL (B) or 120 dB SPL (C). Each point represents the mean (±S.E.M.) of ABR thresholds measured in 5–8 animals, upon completion of the ASR testing period. Results from A, B and C are represented as threshold shifts in D, E and F, respectively. *: p<0.05, **: p<0.01, ***: p<0.001.

In order to assess the impact of sound exposure on hearing function, we first compared mean ABR thresholds in animals exposed at each of the three levels of sound (110, 115 and 120 dB SPL) with those of control animals (Fig. 2A–C). Mean thresholds in control animals varied somewhat across frequencies but ranged between 18 and 33 dB SPL, which proved to be insignificant when comparing across the three control groups (F_2,55_ = 2.32, P = 0.11)(Fig. 2A–C, open circles). The mean threshold in exposed animals varied between 33 and 93 dB, increasing approximately linearly with the level of exposure at all frequencies (R = 0.72, P = 0.0007). The thresholds in exposed animals were significantly higher than those in their respective control groups in all three exposure level comparisons (F_1,31_ = 53.64, P<0.0001 for the 110 dB SPL exposure group, F_1,35_ = 50.08, P<0.0001 for the 115 dB SPL exposure group, and F_1,51_ = 165.3, P<0.001 for the 120 dB SPL exposure group). Maximal threshold shifts in exposed animals were consistently at 8 and 12 kHz and measured 36–38 dB, 49–52 dB and 60–76 dB for the 110, 115 and 120 dB SPL exposure groups, respectively (Fig. 2D–F). The number of frequencies at which significant threshold shifts occurred, as well as the extent of the shift, also increased with exposure level: 8 and 12 kHz in the 110 dB SPL group (P = 0.007 and 0.04, respectively), 8 and 12 kHz in the 115 dB SPL exposure group (P = 0.006 for both), and all four test frequencies (4, 8, 12 and 16 kHz) in the 120 dB SPL group (P values, 0.003, 0.0006, 0.0004 and 0.01, respectively). Thus, both the degree of threshold shift and the spectral range of these shifts increased with the level of exposure.

### Effect of post-exposure recovery time on ASR

**Figure 3:**
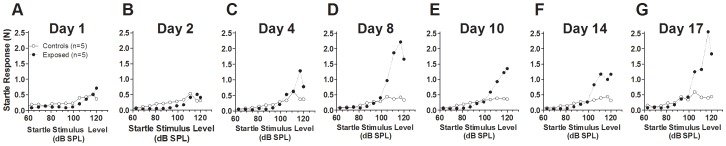
Comparison of the ASR growth curves from control (n = 5) and exposed animals (n = 6) at each of the 7 post-exposure times indicated at the top of each graph (panels A–G). The data shows decrements in ASR in exposed animals during the first two days post-exposure, but by the fourth day, there is a suggestion of enhanced ASR at the highest startle stimulus levels. This enhancement was better established by the 8^th^ day at all stimulus levels above 100 dB SPL and continued through the remainder of the 2 weeks of measurements, although the degree of enhancement varied over time.

Changes in the amplitudes of the ASR displayed considerable plasticity and rebound following tone exposure. The period of most dynamic change was the first few days following exposure, but additional quantitative changes continued through the remainder of the 3 or more weeks of measurements. The mean ASR growth curves for animals exposed at 115 dB SPL are shown in Fig. 3. Here, startle amplitudes can be seen to be either similar to or slightly diminished below control levels during the first 2 days following exposure (Fig. 3A–B). However, between 4 and 8 days post-exposure, a trend towards enhanced startle amplitudes were clearly evident at high levels of stimulation (Fig. 3C–D), and these enhancements continued to be apparent, albeit with some fluctuation, throughout the period of testing (Fig. 3E–G). The startle enhancements observed 1 week following exposure are similar to those described in our previous paper, which was based on a different set of animals [18], but the time series of Fig. 3 shows for the first time that the enhancement was a secondary effect that took several days to develop, suggesting the involvement of plastic mechanisms that are triggered by the initial insult.

**Figure 4:**
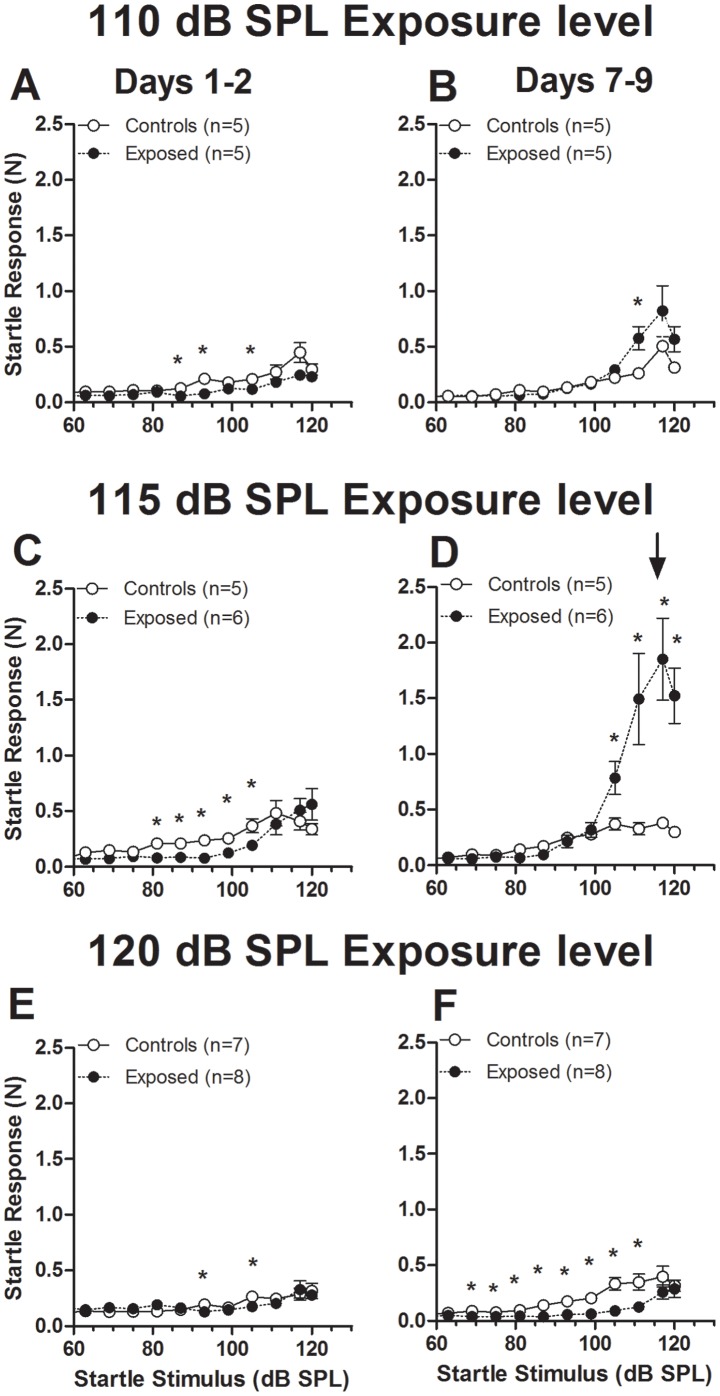
Effects of exposure level on ASR growth curves for early (1–2 days) and late (7–9 days) post-exposure time frames. A–B. Data for animals exposed at 110 dB SPL. C–D. Data for animals exposed at 115 dB SPL. E–F. Data for animals exposed at 120 dB SPL. Each point represents the mean (±S.E.M.). Group sizes are indicated in the graphs. Asterisks indicate points where differences between exposed and control animals were statistically significant (p<0.05).

ASR enhancements of the type shown in Fig. 3 were characteristic of most animals exposed at 115 dB SPL and some animals exposed at 110 dB SPL (Fig. 4, top and middle rows), although the precise details varied somewhat in the absolute amplitudes of startle and in the time course of the changes. When the data were averaged across animals on days 7–9 post-exposure, those exposed at 110 showed a trend suggestive of startle enhancements at high startle stimulus levels (≥105 dB SPL) (F_3,72_ = 6.21, P = 0.01), but the enhancement was significant only at a startle stimulus level of 110 dB SPL (T_9_ = 1.83, P = 0.05) (Fig. 4B). However, in the animals exposed at 115 dB SPL, the increases in ASR amplitude were highly significant at all stimulus levels from 105–120 dB SPL (Fig. 4D) (F_3,80_ = 38.57, P<0.0001) and followed an initial period when startle responses were decreased below control levels (Fig. 4C). Post hoc t tests yielded P values no higher than 0.01 ( = 2.55, *P*<0.01 at 105 dB SPL, *T_9_* = 2.63, *P*<0.01 at 110 dB SPL, *T_9_ = *3.38, *P*<0.005 at 115 dB SPL and *T_9_ = *4.83, *P*<0.001 at 120 dB SPL*).*

**Figure 5:**
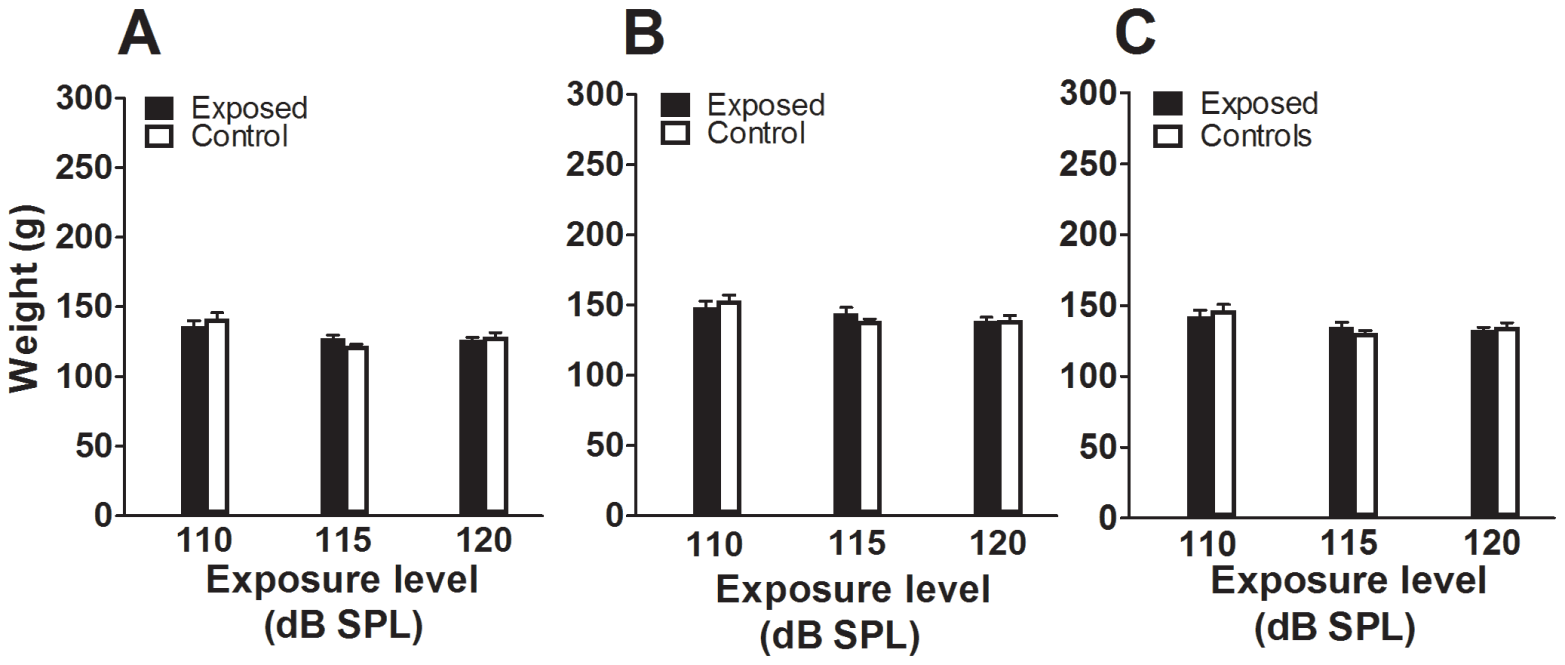
Representation of control and exposed animal weights, by group, from the beginning to the end of the ASR testing period. A. Weight averages on the first day of ASR testing, which is also the first day post-exposure. B. Weight averages on the last day of ASR testing. C. Weight averages over time through the entire ASR testing period. None of these measurements showed significant differences between exposed and control animals. (All P values >0.05, see text for exact values).

An altogether different pattern of change was observed in animals exposed at 120 dB SPL. In that group, both startle responses and the baseline level of activity below the startle threshold were below those of controls; moreover, the initial weakening of ASR amplitude observed above startle threshold on the first and second day after exposure (Fig. 4E) persisted and became even more pronounced when the same measures were performed 7–9 days after exposure (Fig. 4F). The decreases in ASR amplitude in this group of exposed animals were pronounced when tested by two-way ANOVA (F_12,364_ = 45.66, P<0.001), with significant differences found at all startle stimulus levels from 70 to 110 dB SPL (P values for points represented by asterisks in Fig. 4F ranged from 0.000007 at 93 dB SPL to 0.02 at 110 dB SPL). Thus, our results show that the effect of exposure on ASR growth curves is dependent not only on the level of the exposure but also on the intensity of the startle-eliciting stimulus and the time after exposure when the measures are performed. None of the changes in ASR amplitude could be attributed to difference in weight of the animals. As shown in Fig. 5, there were no significant differences between mean weights in exposed and control animals at the beginning (T_4_ = 1.05, P = 0.35 for the 110 dB SPL group, T_5_ = 1.81, P = 0.13 for the 115 dB SPL group, and T_6_ = 0.99, P = 0.36 for the 120 dB SPL group) and end (T_4_ = 1.02, P = 0.37 for the 110 dB SPL group, T_5_ = 1.13, P = 0.31 for the 115 dB SPL group, and T_6_ = 0.33, P = 0.75 for the 120 dB SPL group) of the ASR testing periods (Fig. 5A–B). Moreover, there was no significant difference between mean weights in exposed and control animals when averaged over time through the period of ASR testing (T_4_ = 0.85, P = 0.44 for the 110 dB SPL group, T_5_ = 1.17, P = 0.29 for the 115 dB SPL group, and T_6_ = 0.99, P = 0.36 for the 120 dB SPL group) (Fig. 5C).

### ASR amplitude and threshold shift

**Figure 6:**
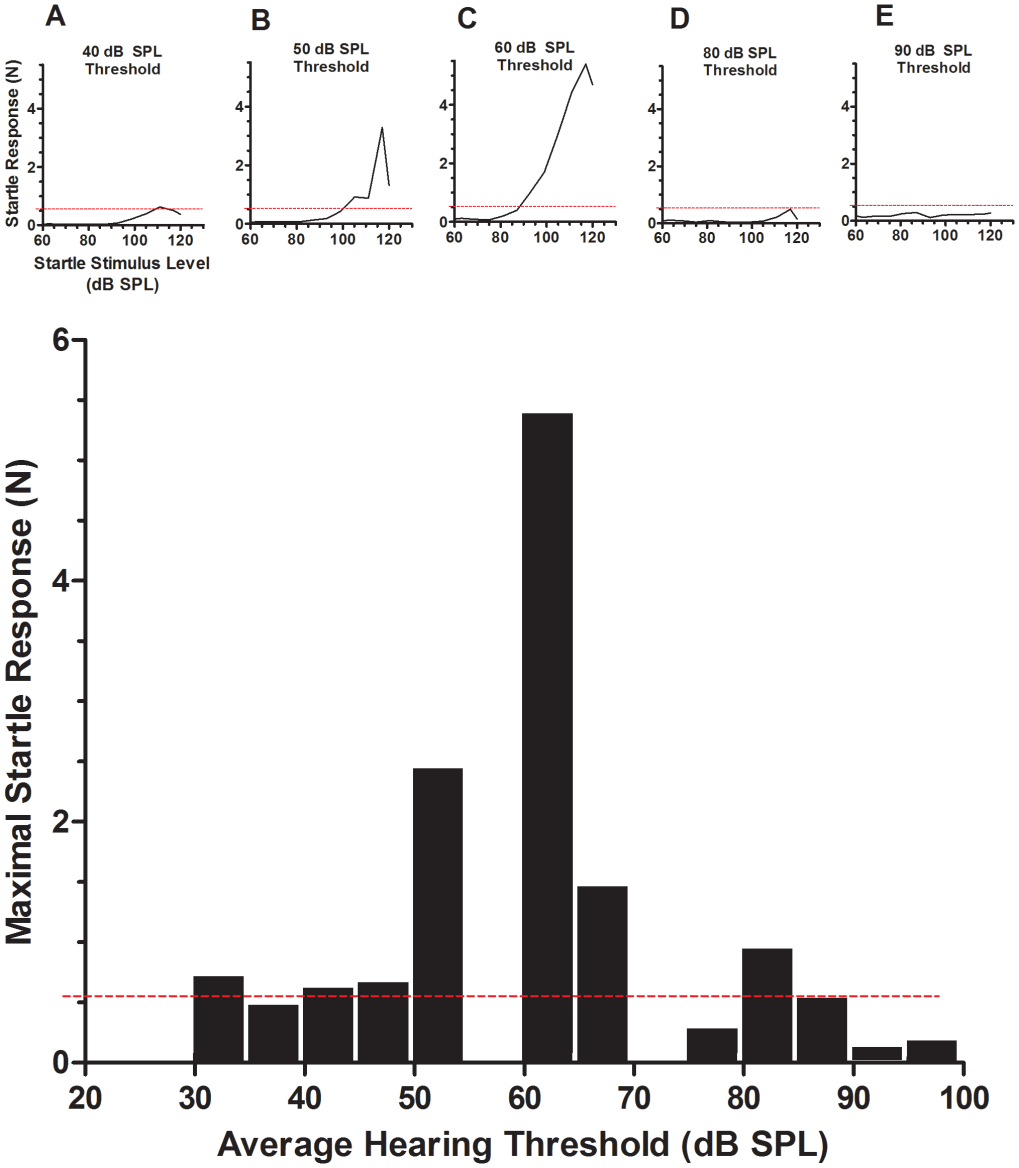
Dependence of enhancement of startle on threshold. The histogram depicts maximal ASR amplitudes with respect to thresholds for exposed animals. The dashed red line represents the mean maximal startle amplitude of control animals (thresholds for these animals ranged from 18 to 33 dB SPL). A–E: Representative ASR growth curves of exposed animals with different thresholds. When thresholds were less than 50 dB, startle amplitudes were similar to those of controls. For animals with thresholds of 50–70 dB, enhancements of startle were clearly apparent, but for animals with thresholds above 70 dB SPL, ASR amplitudes were in most cases reduced below control levels.

The data in Fig. 4 suggests a link between the magnitude of change in the ASR and exposure level, but they obscure the more important relationship of how the change in ASR relates to the post-exposure thresholds. To examine this relationship, we plotted the maximal ASR amplitude as a function of average threshold, irrespective of the level of sound to which the animals were exposed. The results in Fig. 6 show that the enhancement of startle was highly dependent on the loss in sound sensitivity, being limited to a narrow range of thresholds between 50 and 70 dB SPL. Below this range (30–50 dB SPL), startle amplitudes were comparable to control levels, but above this range (80–100 dB SPL), ASR amplitudes declined toward sub-control values with further increases in threshold. Thus, moderate threshold elevation restricted to a narrow range resulted in ASR enhancements, whereas severe threshold elevation resulted in a weakening of ASR.

### Effect of acoustic context

**Figure 7:**
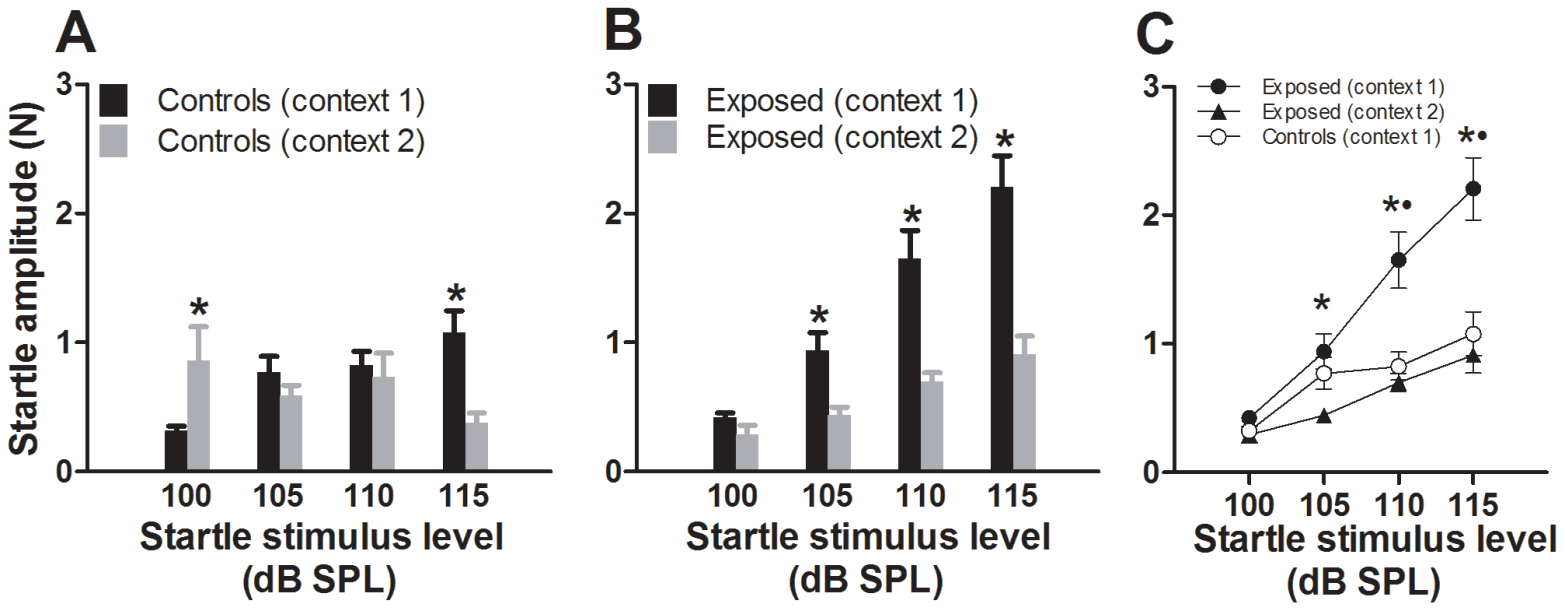
Effect of context on startle amplitudes. A. Data for control animals. B. Data for exposed animals. In each graph, data from animals tested in the context of startle stimuli alone (context 1) are compared with data from animals tested using startle stimuli alone presented randomly in the context of the gap detection test (context 2). Note that in control animals, ASR amplitudes were generally weaker in context 2 than in context 1, but for the most part, the decreases were not significant. In exposed animals, ASRs measured in context 2 were consistently weaker than those measured in context 1, indicating an effect of context on startle suppression. C. Comparison of startle amplitudes of exposed animals tested in contexts 1 and 2 with startle amplitudes of controls tested in context 1, showing the loss of hyperresponsiveness when animals were tested in context 2. *: P<0.05 for comparison between exposed animals tested in the two contexts. Dot represents P<0.05 for comparison between exposed and controls tested in context 1.

The results of this experiment are shown in Fig. 7. Control animals showed context-dependent differences in startle amplitudes at the low and high startle stimulus levels (Fig. 7A). The exposed animals showed even more pronounced context-dependent differences, startle amplitudes being consistently lower in the context of the gap suppression test (context 2) than in the context of the startle growth curve test (context 1) (Fig. 7B); also, exposed animals showed a trend towards increasingly larger context-dependent differences with increases in stimulus level. Significantly weaker startle amplitudes were found in exposed animals at three of the four startle stimulus levels in context 2 than in context 1 (F_2,16_ = 5.34, P = 0.02 at 105 dB SPL, F_2,16_ = 10.44, P = 0.013 at 110 dB SPL and F = 13.03, P = 0.0004 at 115 dB SPL). Only in the condition where the startle stimulus level was 100 dB SPL were ASRs in exposed animals similar in the two contexts. Moreover, relative to controls, the significance of the enhancements of startle observed in exposed animals in context 1 (F_1,64_ = 27.02, P<0.0001), was not observed in context 2 (Fig. 7C). In fact, the ASR obtained in context 2 were consistently lower than those obtained from control animals tested in context 1 (F_1,64_ = 4.74, P<0.05). This indicates that the more complex stimulus battery of the gap detection test was suppressive of the ASR in both groups, although the suppression was more consistent and level dependent than in controls.

## Discussion

Our results show that the direction and magnitude of change in the ASR is dependent on numerous factors, including the exposure level, the degree of threshold shift, the intensity of stimulation, the time after exposure when the ASR is measured, and on the context in which the startle stimuli are presented. We now discuss the possible underlying mechanisms of these changes and place our findings into a broader context by comparing them to those reported in other studies.

### Enhanced startle responses were observed only in animals showing moderately elevated threshold

Animals with moderate thresholds (i.e., those in the range of 50–70 dB SPL) displayed robust enhancements of the ASR at stimulus intensities of 110–120 dB SPL. No enhancements were observed in animals with thresholds lower than 50 dB SPL or above 70 dB SPL. The enhanced startle responses observed in animals with thresholds in the range of 50–70 dB SPL emerged with a delayed onset of a few days and followed an initial period during which the ASR was either unchanged or slightly weakened.

One possible explanation of the enhanced ASR in sound exposed animals is that it was the result of Pavlovian conditioning or sensitization of the motor response caused by stress experienced during the initial sound exposure. Several of our observations argue against this explanation. First, we monitored the animals during the exposure periods and observed no evidence of a stressful response to the exposure sound. Animals moved about on the floor of their enclosures seemingly indifferent to the presence of sound. The absence of any apparent stress response could be the result of a decrease in sensation level which the animals would experience as the hearing thresholds were shifted upward as the tone level was gradually stepped up in the beginning of the exposure period. Second, we observed no enhancement of ASR on the days immediately after the exposure, when the memory of the exposure would have been strongest. The enhanced startle responses observed in animals with thresholds in the range of 50–70 dB SPL emerged with a delayed onset of a few days and followed an initial period during which the ASR was either unchanged or slightly weakened. And third, ASR amplitudes were much weaker in the animals with the severest threshold losses, even after correcting for differences in sensation levels (see below). None of these observations seems consistent with the interpretation that animals exposed to intense sound developed enhanced ASRs because of a stress-associated conditioning. This conclusion is further supported by two other recent studies reporting chronically enhanced ASR in animals that were exposed while unconscious due to induction of anesthesia [17], [27].

The enhanced ASR seems more likely to involve plastic mechanisms whereby the gain of the ASR at high stimulus levels is gradually readjusted in response to moderate hearing loss, as suggested in previous studies [13], [18], [28], [29]. Although our data do not reveal mechanisms directly, we can offer some useful speculations concerning underlying mechanisms by considering knowledge based on previous studies. Those studies have shown that the main circuit that mediates the ASR includes cochlear root neurons (CRNs), which project to the caudal pontine reticular nucleus (cPRN), which, in turn, project to spinal motoneurons controlling body musculature [30]–[37]. A readjustment of gain of this startle circuit is likely to involve modulation by inputs to one or more of these nuclei from other sources. While such inputs come from multiple sources [30], the dorsal cochlear nucleus (DCN) is of special interest here because it has been shown to play a role in modulating the gain of the high intensity (110–115 dB SPL) component of the ASR [38], approximately the same range of intensities over which enhanced startle responses were observed in the present study. Electrical stimulation of the DCN evokes large monosynaptic EPSPs in the giant neurons of the PRN [39], which activate the spinal motoneurons that recruit the muscles of the acoustic startle reflex [37]. Moreover, tracer injections into the giant cell regions of the PRN resulted in retrograde labeling of cochlear root neurons in the VCN and large neurons (probably fusiform and/or giant cells) in the DCN [35], [37]. Thus, there are both physiological and anatomical grounds on which to speculate that enhancements of startle might result from hyperactivity of output neurons in the DCN that synapse on the giant neurons of the PRN. Fusiform cells have been found to become hyperactive after intense sound exposure [40]–[43]. A number of studies suggest that noise exposure causes shifts in the balance of excitation and inhibition towards the side of excitation of DCN fusiform cells [44]–[46]. Increased activity of these cells would be expected to increase input to the giant cells of the PRN, thus increasing the amplitude of the ASR. Other sources of input to the acoustic startle circuit could also be involved, although thus far, none of these has been shown to play a role in adjusting the gain of the high intensity component of the acoustic startle reflex.

### The ASR was weakened in animals with severe threshold shift

A fundamental question raised by our results is why, despite the induction of enhanced ASR with moderate thresholds (50–70 dB SPL), severely raised thresholds (those above 70 dB SPL) were associated with a persistent decrement of the ASR. Previous studies indicate that weakening of inhibition and strengthening of excitation can be induced in central auditory nuclei for different degrees (moderate and severe) of hearing loss [44], [46]. Thus, to explain the decrement of the ASR observed in animals with the highest thresholds requires additional contributions via mechanisms that are not triggered by moderate hearing loss. The lack of enhancement of ASR in animals with thresholds greater than 70 dB SPL may be due in large part to the more severe hearing loss itself. Because sensation levels of high level startle stimuli would mostly be lower in animals with high thresholds than in those with moderate thresholds, the ASR would be expected to be correspondingly weaker. On the other hand, it seems unlikely that hearing loss can completely explain the lack of enhanced ASR in the highest threshold group. Weaker ASRs were found in this group even when comparing ASR amplitudes at startle stimulus levels evoking similar sensation levels. For example, using the highest ABR thresholds of Fig. 2B and 2C as benchmarks (80 dB SPL in animals exposed at 115 dB SPL and 93 dB SPL in the animals exposed at 120 dB SPL), startle stimuli at 25–30 dB SL would be in the range between 105 and 110 dB SPL for animals exposed at 115 dB SPL (Fig. 4D) and between 110 and 115 dB SPL for animals exposed at 120 dB SPL (Fig. 4F). Comparison of ASR amplitudes at these similar sensation levels still reveals a weaker response in the 120 dB group than those exposed at 115 dB group. Thus, other mechanisms would appear to contribute to the weakened ASR in the high threshold group.

One such mechanism may be the anatomical loss of primary afferent input to CRNs. Moderate levels of exposure (i.e., up to 110 dB SPL) typically causes loss of outer hair cells and injury to inner hair cells [47]–[49] as well as excitotoxic injury to the peripheral dendrites of spiral ganglion neurons [19], [20], [50]. This leaves surviving spiral ganglion cells and their centrally extending axons spontaneously inactive and unresponsive to sound [51], [52], diminishing functional but not anatomical input to recipient CRN neurons. In contrast, higher levels of exposure cause more severe damage with widespread loss of outer and inner hair cells [47], [53]. A secondary consequence of this type of injury is trans-neuronal degeneration of auditory nerve fibers which can spread to recipient neurons in the VCN [19], [54]–[56]. Since CRNs receive direct input from primary afferents [57]–[60], this could lead to transneuronal degeneration of PRNs, irreversibly reducing the gain of the ASR, despite the survival of other cochlear nucleus cell types that may become hyperactive and also project to PRNs. Future studies examining the effects of severe acoustic insult on PRNs are needed to test this hypothesis more directly.

### The present results may explain some of the differing effects of sound exposure on the ASR reported previously

One of the factors motivating our study was the puzzling discrepancies across studies describing the effects of sound exposure on the ASR. Whereas our previous study [18] and some others have shown chronic enhancement of the ASR in animals previously exposed to intense sound [19], [27], the degrees and time courses of those enhancements as well as the types of stimulus batteries in which they have been observed have differed markedly across studies. Sun et al. [16] reported enhanced ASR immediately following noise exposure, but the enhancement was short lived, disappearing within the first 24 hours following exposure. The disappearance of enhanced ASR within the first 24 hours is not inconsistent with our results showing a lack of enhanced startle in animals tested 1–2 days post-exposure. Hickox and Liberman [19] found enhancements of ASR in animals tested with noise bursts against background noise, but only weak enhancements when tested with noise bursts in silence. This result is similar to the slight enhancement of startle observed in the present study in animals exposed at 110 dB SPL. The enhancement observed in silence by Hickox and Liberman might have been weak because the exposure level was only 100 dB SPL, inner hair cells were spared, and ABR thresholds were not permanently shifted. As our results from animals with near normal ABR thresholds show (Fig. 6), this would have been insufficient to cause robust enhancements of the ASR in the absence of background noise.

Two investigations have presented data showing reductions of ASRs after noise exposure. The reductions in these studies followed 1 hour exposures at levels between 115 and 120 [21] or between 120 and 126 dB SPL [22]. Although the amounts of threshold shift or injury to the ear were not reported, exposure sounds in both studies were presented to animals under anesthesia, a manipulation which could have increased injury to the inner ear by removing the protective effect of the middle ear muscles. Thus, the reductions in the latter two studies could have been a consequence of severely elevated thresholds (>70 dB), similar to the decrements of ASR which we observed in animals exposed at 120 dB SPL.

### The response to a startle stimulus depends on the context

A surprising result of our experiments was the context dependency of the enhancement effect of exposure on ASR amplitude. We found that in exposed animals, when the ASR amplitudes were extracted from the startle-only stimulus conditions embedded in the gap detection test, the enhancement of startle was not observed. Context-dependent differences were also observed in control animals, but these were not as striking. These results are significant for at least two reasons. First, they underscore the importance of separating tests of startle growth functions from gap detection tests if enhancements of startle caused by noise exposure are the focus of investigation. As our results show, none of the startle amplitudes at any of the startle stimulus levels tested from 100–115 dB SPL, the range in which the startle amplitudes were enhanced when the startle stimuli were tested separately, was significantly elevated above control levels when such stimuli were presented in the context of the gap detection test. Second, they suggest that one consequence of sound exposure is a strengthening of suppressive mechanisms, which are either weak or absent in normal hearing animals. This is somewhat unexpected because generally, as discussed above, sound exposures are thought to cause weakening of inhibition and strengthening of excitation [61]. While the enhanced startle observed in our animals is consistent with this, the loss of that enhancement when the ASR is tested in the context of the gap detection test, suggests that the presence of the background noise or some other cue contained in the gap detection test battery elicits a suppression of startle at high stimulus levels not seen in controls. These results may be related to two other forms of enhanced suppression observed in noise exposed animals, including enhanced prepulse inhibition [15], [17] and increased suppression of the ASR by background noise [18]. The results in the present study suggest that noise exposure might increase the duration of the suppressive effect of background noise on the ASR. Whether these effects reflect an increase in the strength of inhibitory synapses [62] or changes in other non-synaptic mechanisms (e.g., adaptation, short term depression) is a topic for future investigation.

### Are enhancements of the ASR related to hyperacusis?

Although the term ‘hyperacusis’ is most widely used in clinical audiology to refer to diminished sound tolerance [4], [9], the underlying basis of diminished sound tolerance is unknown. One possibility is that it reflects a change in emotional sensitivity to sound, irrespective of a change in the sound sensation itself. Alternatively, diminished sound tolerance may result from heightened sense of loudness. Indeed, many clinicians measure hyperacusis either by assessing loudness discomfort level or by directly assessing loudness itself (magnitude estimation of loudness) or some emotional or behavioral quantity that increases with loudness, such as annoyance or fear [63]. As discussed recently in an in-depth review [5], many clinicians use the term ‘hyperacusis’ broadly to include any abnormally heightened percept evoked by sound, including not only loudness, but also increased responsiveness (hyperresponsiveness) to sound [3], [29], [64]. If indeed hyperacusis is a state of enhanced loudness, then one would expect that other quantities that vary with loudness, such as behavioral or physiological responses to sound, would also be enhanced. Evidence that this is true comes from functional imaging studies in humans with hyperacusis [2], [65]. It follows that any circuit connected to the gain of the auditory system which underlies this increased responsiveness to sound, including the acoustic startle reflex circuit, might also show enhancement [28]. These are likely to be reasons why the terms hyperacusis or ‘hyperacusis-like’ are commonly used to refer to measures demonstrative of hyperresponsiveness to sound [11]–[14], [17]–[19], [28], [63]. However, it should be acknowledged that until studies demonstrate that enhanced acoustic startle reflexes are a common characteristic of patients diagnosed with hyperacusis, the term ‘hyperacusis’ should be used with caution in seeking to establish animal models of this condition.

## References

[1] Fournier P, Hébert S (2013) Gap detection deficits in humans with tinnitus as assessed with the acoustic startle paradigm: Does tinnitus fill in the gap? Hear. Res. 295: 16–23. 10.1016/j.heares.2012.05.011 22688322

[2] Gu JW, Halpin CF, Nam EC, Levine RA, Melcher JR (2010) Tinnitus, diminished sound-level tolerance, and elevated auditory activity in humans with clinically normal hearing sensitivity. J. Neurophysiol. 104: 3361–3370. 10.1152/jn.00226.2010 PMC3007631 20881196

[3] Dauman R, Bouscau-Faure F (2005) Assessment and amelioration of hyperacusis in tinnitus patients. Acta Otolaryngol. 125: 503–509. 10.1080/00016480510027565 16092541

[4] Baguley DM (2014) Hyperacusis: An Overview. Sem Hearing. 35: 4–83.

[5] Tyler RS, Pienkowski M, Rojas Roncancio E, Jun HJ, Brozoski T, et al (2014) Review of Hyperacusis and Future Directions: Part I. Definitions and Manifestations. Am J Audiol. Aug 7: AJA–14-0010. 10.1044/2014_AJA-14-0010 25104073

[6] Jastreboff PJ, Hazell JWP (2004) Tinnitus Retraining Therapy: Implementing the neurophysiological model. Cambridge University Press. 1–276.

[7] Andersson G, Lindvall N, Hursti T, Carlbring P (2002) Hypersensitivity to sound (hyperacusis): a prevalence study conducted via the Internet and post. Int J Audiol. 41(8): 545–54. 10.3109/14992020209056075 12477175

[8] Rubinstein B (1993) Tinnitus and craniomandibular disorders-is there a link? Swed Dent J Suppl. 95: 1–46. 8503098

[9] Baguley DM (2003) Hyperacusis. J R Soc Med. 96(12): 582–5. 10.1258/jrsm.96.12.582 PMC539655 14645606

[10] Schecklmann M, Landgrebe M, Langguth B (2014) TRI Database Study Group (2014) Phenotypic characteristics of hyperacusis in tinnitus. PLoS One. 9(1): e86944. 10.1371/journal.pone.0086944 PMC3908961 24498000

[11] Ison JR, Allen PD, O'Neill WE (2007) Age-related hearing loss in C57BL/6J mice has both frequency-specific and non-frequency-specific components that produce a hyperacusis-like exaggeration of the acoustic startle reflex. J. Assoc. Res. Otolaryngol. 8: 539–550. 10.1007/s10162-007-0098-3 PMC2538342 17952509

[12] Turner JG, Parrish J (2008) Gap detection methods for assessing salicylate-induced tinnitus and hyperacusis in rats. Am. J. Audiol. 17: S185–192. 10.1044/1059-0889(2008/08-0006) 18978200

[13] Sun W, Lu J, Stolzberg D, Gray L, Deng A, et al (2009) Salicylate increases the gain of the central auditory system. Neuroscience. 159(1): 325–34. 10.1016/j.neuroscience.2008.12.024 PMC2759817 19154777

[14] Lu J, Lobarinas E, Deng A, Goodey R, Stolzberg D, et al (2011) GABAergic neural activity involved in salicylate-induced auditory cortex gain enhancement. Neuroscience. 189: 187–98. 10.1016/j.neuroscience.2011.04.073 PMC3153886 21664433

[15] Turner J, Larsen D, Hughes L, Moechars D, Shore S (2012) Time course of tinnitus development following noise exposure in mice. J Neurosci Res. 90(7): 1480–8. 10.1002/jnr.22827 PMC3725635 22434653

[16] Sun W, Deng A, Jayaram A, Gibson B (2012) Noise exposure enhances auditory cortex responses related to hyperacusis behavior. Brain Res. 1485: 108–16. 10.1016/j.brainres.2012.02.008 22402030

[17] Dehmel S, Eisinger D, Shore SE (2012) Gap prepulse inhibition and auditory brainstem-evoked potentials as objective measures for tinnitus in guinea pigs. Front Syst Neurosci. 6: 42. 10.3389/fnsys.2012.00042 PMC3364697 22666193

[18] Chen G, Lee C, Sandridge SA, Butler HM, Manzoor NF, et al (2013) Behavioral evidence for possible simultaneous induction of hyperacusis and tinnitus following intense sound exposure. J Assoc Res Otolaryngol. 14(3): 413–24. 10.1007/s10162-013-0375-2 PMC3642276 23440516

[19] Hickox AE, Liberman MC (2014) Is noise-induced cochlear neuropathy key to the generation of hyperacusis or tinnitus? J Neurophysiol. 111(3): 552–64. 10.1152/jn.00184.2013 PMC3921399 24198321

[20] Kujawa SG, Liberman MC (2009) Adding insult to injury: cochlear nerve degeneration after “temporary” noise-induced hearing loss. J Neurosci. 29(45): 14077–85. 10.1523/JNEUROSCI.2845-09.2009 PMC2812055 19906956

[21] Longenecker RJ, Galazyuk AV (2012) Methodological optimization of tinnitus assessment using prepulse inhibition of the acoustic startle reflex. Brain Res. 1485: 54–62. 10.1016/j.brainres.2012.02.067 22513102

[22] Lobarinas E, Hayes SH, Allman BL (2013) The gap-startle paradigm for tinnitus screening in animal models: limitations and optimization. Hear Res. 295: 150–60. 10.1016/j.heares.2012.06.001 PMC3505812 22728305

[23] Carlson S, Willott JF (2001) Modulation of the Acoustic Startle Response by Background Sound in C57BL/6J Mice. In: Willott JF, editor. Handbook of mouse auditory research: from behavior to molecular biology. CRC Press. Pp. 83–90.

[24] Gerrard RL, Ison JR (1990) Spectral frequency and the modulation of the acoustic startle reflex by background noise. J Exp Psychol Anim Behav Process. 16(1): 106–12. 2303789

[25] Ison JR, Hammond GR, Krauter EE (1973) Effects of experience on stimulus-produced reflex inhibition in the rat. J Comp Physiol Psychol. 83(2): 324–36. 10.1037/h0034423 4706596

[26] Ison JR, Taylor MK, Bowen GP, Schwarzkopf SB (1997) Facilitation and inhibition of the acoustic startle reflex in the rat after a momentary increase in background noise level. Behav Neurosci. 111(6): 1335–52. 10.1037//0735-7044.111.6.1335 9438802

[27] Pace E, Zhang J (2013) Noise-induced tinnitus using individualized gap detection analysis and its relationship with hyperacusis, anxiety, and spatial cognition. PLoS One. 8(9): e75011. 10.1371/journal.pone.0075011 PMC3771890 24069375

[28] Zeng FG (2013) An active loudness model suggesting tinnitus as increased central noise and hyperacusis as increased nonlinear gain. Hear Res. 295: 172–9. 10.1016/j.heares.2012.05.009 PMC3593089 22641191

[29] Hébert S, Fournier P, Noreña A (2013) The auditory sensitivity is increased in tinnitus ears. J Neurosci. 33: 2356–64. 10.1523/JNEUROSCI.3461-12.2013 PMC6619157 23392665

[30] Davis M, Gendelman DS, Tischler MD, Gendelman PM (1982) A primary acoustic startle circuit: lesion and stimulation studies. J Neurosci. 2(6): 791–805. 10.1523/JNEUROSCI.02-06-00791.1982 PMC6564345 7086484

[31] Davis M (1984) The mammalian startle response. In: Neural mechanisms of startle behavior. (Eaton RC, ed), 287–351. London: Plenum.

[32] Pellet J (1990) Neural organization in the brainstem circuit mediating the primary acoustic head startle: an electrophysiological study in the rat. Physiol Behav. 48(5): 727–39. 10.1016/0031-9384(90)90218-s 2082373

[33] Lee Y, López DE, Meloni EG, Davis M (1996) A primary acoustic startle pathway: obligatory role of cochlear root neurons and the nucleus reticularis pontis caudalis. J Neurosci. 16(11): 3775–89. 10.1523/JNEUROSCI.16-11-03775.1996 PMC6578836 8642420

[34] Kandler K, Herbert H (1991) Auditory projections from the cochlear nucleus to pontine and mesencephalic reticular nuclei in the rat. Brain Res. 562(2): 230–42. 10.1016/0006-8993(91)90626-7 1773339

[35] Lingenhöhl K, Friauf E (1994) Giant neurons in the rat reticular formation: a sensorimotor interface in the elementary acoustic startle circuit? J Neurosci. 14(3 Pt 1): 1176–94. 10.1523/JNEUROSCI.14-03-01176.1994 PMC6577542 8120618

[36] López DE, Saldaña E, Nodal FR, Merchán MA, Warr WB (1999) Projections of cochlear root neurons, sentinels of the rat auditory pathway. J Comp Neurol. 415(2): 160–74. 10545157

[37] Nodal FR, López DE (2003) Direct input from cochlear root neurons to pontine reticulospinal neurons in albino rat. J Comp Neurol. 460(1): 80–93. 10.1002/cne.10656 12687698

[38] Meloni EG, Davis M (1998) The dorsal cochlear nucleus contributes to a high intensity component of the acoustic startle reflex in rats. Hear Res. 119(1–2): 69–80. 10.1016/s0378-5955(98)00040-9 9641320

[39] Lingenhöhl K, Friauf E (1992) Giant neurons in the caudal pontine reticular formation receive short latency acoustic input: an intracellular recording and HRP-study in the rat. J Comp Neurol. 325(4): 473–92. 10.1002/cne.903250403 1281843

[40] Brozoski TJ, Bauer CA, Caspary DM (2002) Elevated fusiform cell activity in the dorsal cochlear nucleus of chinchillas with psychophysical evidence of tinnitus. J Neurosci. 22(6): 2383–90. 10.1523/JNEUROSCI.22-06-02383.2002 PMC6758251 11896177

[41] Finlayson PG, Kaltenbach JA (2009) Alterations in the spontaneous discharge patterns of single units in the dorsal cochlear nucleus following intense sound exposure. Hear Res. 256(1–2): 104–17. 10.1016/j.heares.2009.07.006 PMC2778575 19622390

[42] Li S, Choi V, Tzounopoulos T (2013) Pathogenic plasticity of Kv7.2/3 channel activity is essential for the induction of tinnitus. Proc Natl Acad Sci U S A. 110(24): 9980–5. 10.1073/pnas.1302770110 PMC3683764 23716673

[43] Shore SE, Koehler S, Oldakowski M, Hughes LF, Syed S (2008) Dorsal cochlear nucleus responses to somatosensory stimulation are enhanced after noise-induced hearing loss. Eur J Neurosci. 27(1): 155–68. 10.1111/j.1460-9568.2007.05983.x PMC2614620 18184319

[44] Asako M, Holt AG, Griffith RD, Buras ED, Altschuler RA (2005) Deafness-related decreases in glycine-immunoreactive labeling in the rat cochlear nucleus. J Neurosci Res. 81(1): 102–9. 10.1002/jnr.20542 PMC4455948 15929063

[45] Dong S, Mulders WH, Rodger J, Robertson D (2009) Changes in neuronal activity and gene expression in guinea-pig auditory brainstem after unilateral partial hearing loss. Neuroscience. 159(3): 1164–74. 10.1016/j.neuroscience.2009.01.043 19356697

[46] Wang H, Brozoski TJ, Turner JG, Ling L, Parrish JL, et al (2009) Plasticity at glycinergic synapses in dorsal cochlear nucleus of rats with behavioral evidence of tinnitus. Neuroscience. 164(2): 747–59. 10.1016/j.neuroscience.2009.08.026 PMC2761999 19699270

[47] Kaltenbach JA, Schmidt RN, Kaplan CR (1992) Tone-induced stereocilia lesions as a function of exposure level and duration in the hamster cochlea. Hear Res. 60(2): 205–15. 10.1016/0378-5955(92)90022-f 1639730

[48] Fredelius L, Johansson B, Bagger-Sjöbäck D, Wersäll J (1987) Qualitative and quantitative changes in the guinea pig organ of Corti after pure tone acoustic overstimulation. Hear Res. 30(2–3): 157–67. 10.1016/0378-5955(87)90133-x 3680063

[49] Emmerich E, Richter F, Linss V, Linss W (2005) Frequency-specific cochlear damage in guinea pig after exposure to different types of realistic industrial noise. Hear Res. 201(1–2): 90–8. 10.1016/j.heares.2004.09.009 15721564

[50] Puel JL, Ruel J, Gervais d'Aldin C, Pujol R (1998) Excitotoxicity and repair of cochlear synapses after noise-trauma induced hearing loss. Neuroreport. 9(9): 2109–14. 10.1097/00001756-199806220-00037 9674603

[51] Liberman MC, Kiang NY (1978) Acoustic trauma in cats. Cochlear pathology and auditory-nerve activity. Acta Otolaryngol Suppl. 358: 1–63. 281107

[52] Liberman MC, Dodds LW (1984) Single-neuron labeling and chronic cochlear pathology. II. Stereocilia damage and alterations of spontaneous discharge rates. Hear Res. 16(1): 43–53. 10.1016/0378-5955(84)90024-8 6511672

[53] Stebbins WC, Hawkins JE Jr, Johnson LG, Moody DB (1979) Hearing thresholds with outer and inner hair cell loss. Am J Otolaryngol. 1(1): 15–27. 10.1016/s0196-0709(79)80004-6 95382

[54] Morest DK, Bohne BA (1983) Noise-induced degeneration in the brain and representation of inner and outer hair cells. Hear Res. 9(2): 145–51. 10.1016/0378-5955(83)90024-2 6833159

[55] Morest DK, Kim J, Bohne BA (1997) Neuronal and transneuronal degeneration of auditory axons in the brainstem after cochlear lesions in the chinchilla: cochleotopic and non-cochleotopic patterns. Hear Res. 103(1–2): 151–68. 10.1016/s0378-5955(96)00172-4 9007582

[56] Kim J, Morest DK, Bohne BA (1997) Degeneration of axons in the brainstem of the chinchilla after auditory overstimulation. Hear Res. 103(1–2): 169–191. 10.1016/s0378-5955(96)00173-6 9007583

[57] Harrison JM, Warr WB, Irving R (1962) Second order neurons in the acoustic nerve. Science. 138: 893–895. 10.1126/science.138.3543.893 13952991

[58] Merchan MA, Collia F, Lopez DE, Saldaña E (1988) Morphology of cochlear root neurons in the rat. J Neurocytol. 17(5): 711–25. 10.1007/BF01260998 2463341

[59] Gómez-Nieto R, Rubio ME, López DE (2008) Cholinergic input from the ventral nucleus of the trapezoid body to cochlear root neurons in rats. J Comp Neurol. 506(3): 452–68. 10.1002/cne.21554 18041785

[60] Sinex DG, López DE, Warr WB (2001) Electrophysiological responses of cochlear root neurons. Hear Res. 158(1–2): 28–38. 10.1016/s0378-5955(01)00293-3 11506934

[61] Roberts LE, Eggermont JJ, Caspary DM, Shore SE, Melcher JR, et al (2010) Ringing ears: the neuroscience of tinnitus. J Neurosci. 30(45): 14972–9. 10.1523/JNEUROSCI.4028-10.2010 PMC3073522 21068300

[62] Dong S, Mulders WH, Rodger J, Woo S, Robertson D (2010) Acoustic trauma evokes hyperactivity and changes in gene expression in guinea-pig auditory brainstem. Eur J Neurosci. 31(9): 1616–28. 10.1111/j.1460-9568.2010.07183.x 20525074

[63] Tyler RS, Noble W, Coelho C, Haskell G, Bardia A (2009) Tinnitus and hyperacusis. In: Handbook of Clinical Audiology, Ed. Lippincott, Williams and Wilkins. New York, 726–738.

[64] Song JJ, De Ridder D, Weisz N, Schlee W, Van de Heyning P, et al (2014) Hyperacusis-associated pathological resting-state brain oscillations in the tinnitus brain: a hyperresponsiveness network with paradoxically inactive auditory cortex. Brain Struct Funct. 219: 1113–28. 10.1007/s00429-013-0555-1 23609486

[65] Weber H, Pfadenhauer K, Stöhr M, Rösler A (2002) Central hyperacusis with phonophobia in multiple sclerosis. Mult Scler. 8: 505–9. 10.1191/1352458502ms814oa 12474992

